# Long non-coding RNA HOTAIR promotes cell migration by upregulating insulin growth factor–binding protein 2 in renal cell carcinoma

**DOI:** 10.1038/s41598-017-12191-z

**Published:** 2017-09-20

**Authors:** Hiromichi Katayama, Keiichi Tamai, Rie Shibuya, Mao Nakamura, Mai Mochizuki, Kazunori Yamaguchi, Sadafumi Kawamura, Tatsuo Tochigi, Ikuro Sato, Takamasa Okanishi, Kunie Sakurai, Wataru Fujibuchi, Yoichi Arai, Kennichi Satoh

**Affiliations:** 1 Division of Cancer Stem Cell, Miyagi Cancer Center Research Institute, Natori, Japan; 2 Division of Molecular and Cellular Oncology, Miyagi Cancer Center Research Institute, Natori, Japan; 3 Department of Urology, Miyagi Cancer Center, Natori, Japan; 4 Department of Pathology, Miyagi Cancer Center, Natori, Japan; 5 Center for iPS Cell Research and Application, Kyoto University, Kyoto, Japan; 6 Department of Urology, Tohoku University Graduate School of Medicine, Sendai, Japan

## Abstract

Renal cell carcinoma (RCC) is one of the most lethal urologic cancers. About one-third of RCC patients already have distal metastasis at the time of diagnosis. There is growing evidence that Hox antisense intergenic RNA (HOTAIR) plays essential roles in metastasis in several types of cancers. However, the precise mechanism by which HOTAIR enhances malignancy remains unclear, especially in RCC. Here, we demonstrated that HOTAIR enhances RCC-cell migration by regulating the insulin growth factor-binding protein 2 (IGFBP2) expression. HOTAIR expression in tumors was significantly correlated with nuclear grade, lymph-node metastasis, and lung metastasis. High HOTAIR expression was associated with a poor prognosis in both our dataset and The Cancer Genome Atlas dataset. Migratory capacity was enhanced in RCC cell lines in a HOTAIR-dependent manner. HOTAIR overexpression accelerated tumorigenicity and lung metastasis in immunodeficient mice. Microarray analysis revealed that IGFBP2 expression was upregulated in HOTAIR-overexpressing cells compared with control cells. The enhanced migration activity of HOTAIR-overexpressing cells was attenuated by IGFBP2 knockdown. IGFBP2 and HOTAIR were co-expressed in clinical RCC samples. Our findings suggest that the HOTAIR-IGFBP2 axis plays critical roles in RCC metastasis and may serve as a novel therapeutic target for advanced RCC.

## Introduction

Renal cell carcinoma (RCC), which accounts for about 3% of all cancers in adults, is the most lethal of all urological malignancies^1^. One-third of RCC patients already have metastases at the time of diagnosis, and 20–30% of patients treated by radical nephrectomy will suffer metastasis or recurrence^2^. The prognosis of metastatic RCC is poor: the median survival is about 13 months^3^. Although recent developments in targeted therapy have improved survival rates for metastatic RCC, most patients still succumb to the disease. Therefore, new therapeutic approaches and prognostic factors are needed to treat advanced RCC.

Although numerous lncRNAs (non-coding RNAs longer than 200 nucleotides)^4^ have been identified as factors in cancer progression and the development and spread of metastases^5^, lncRNAs also regulate a wide variety of cell functions in normal tissue. Since many lncRNAs are differentially expressed in specific organs, tissues, or cancer types, lncRNAs are potential prognostic markers^4^.

Hox antisense intergenic RNA (HOTAIR), a lncRNA that acts as an oncogenic molecule in various types of cancer, is localized to the HOXC gene cluster. HOTAIR interacts with PRC2 (polycomb repressive complex 2) to enhance H3K27 trimethylation, and thereby decreases the expression of a large number of genes^6^. Several groups, including our laboratory, have reported that high HOTAIR expression is correlated with a poor prognosis in several types of cancer, including breast^7^, colorectal^8^, cervical^9^, non-small lung cell^10^, and gastric cancer^11^. However, the underlying mechanism by which HOTAIR is involved in malignancy remains uncertain. Many downstream molecules of HOTAIR have been identified: in breast cancer, HOTAIR increases cancer invasiveness and metastasis in a manner dependent on PRC2^7^. In esophageal squamous cell carcinoma, HOTAIR decreases WIF-1 expression and activates the Wnt/β-catenin signaling pathway, thus promoting cell migration^12^. In cervical cancer, HOTAIR promotes tumor growth and invasion by targeting the Notch pathway^13^. However, there are few reports addressing HOTAIR’s molecular mechanism in RCC.

Insulin growth factor-binding protein 2 (IGFBP2) belongs to a family of six IGF-binding proteins, IGFBP1–6. These proteins bind to IGF1 and IGF2. The IGFBP2 expression is elevated in many cancer types, in both tumor cells and plasma^14–16^. Although conventionally known as the IGF regulatory protein, IGFBP was recently demonstrated to have pro-tumorigenic activity that is independent of IGF signaling in glioma cells: IGFBP2 contributes to cancer progression by enhancing MMP2 (matrix metalloprotease 2) gene transcription and, in turn, tumor-cell invasion^17^. IGFBP2 also binds integrin alpha 5 and activates pathways downstream of integrin, increasing cell motility^18^. Exogenous IGFBP2 promotes glioma-cell proliferation and invasion capability via the ERK pathway, which is activated by integrin β1 signaling^19^. However, it is not known how IGFBP2 is regulated in cancer cells, or whether IGFBP2 has oncogenic activity in RCC.

In this study, we analyzed correlations between HOTAIR expression and clinical characteristics in 64 RCCs. We clarified HOTAIR’s role in RCC and identified IGFBP2 as a molecule downstream of HOTAIR that is involved in RCC migratory capacity and prognosis.

## Results

### HOTAIR expression and clinicopathological characteristics in RCC

**Figure 1:**
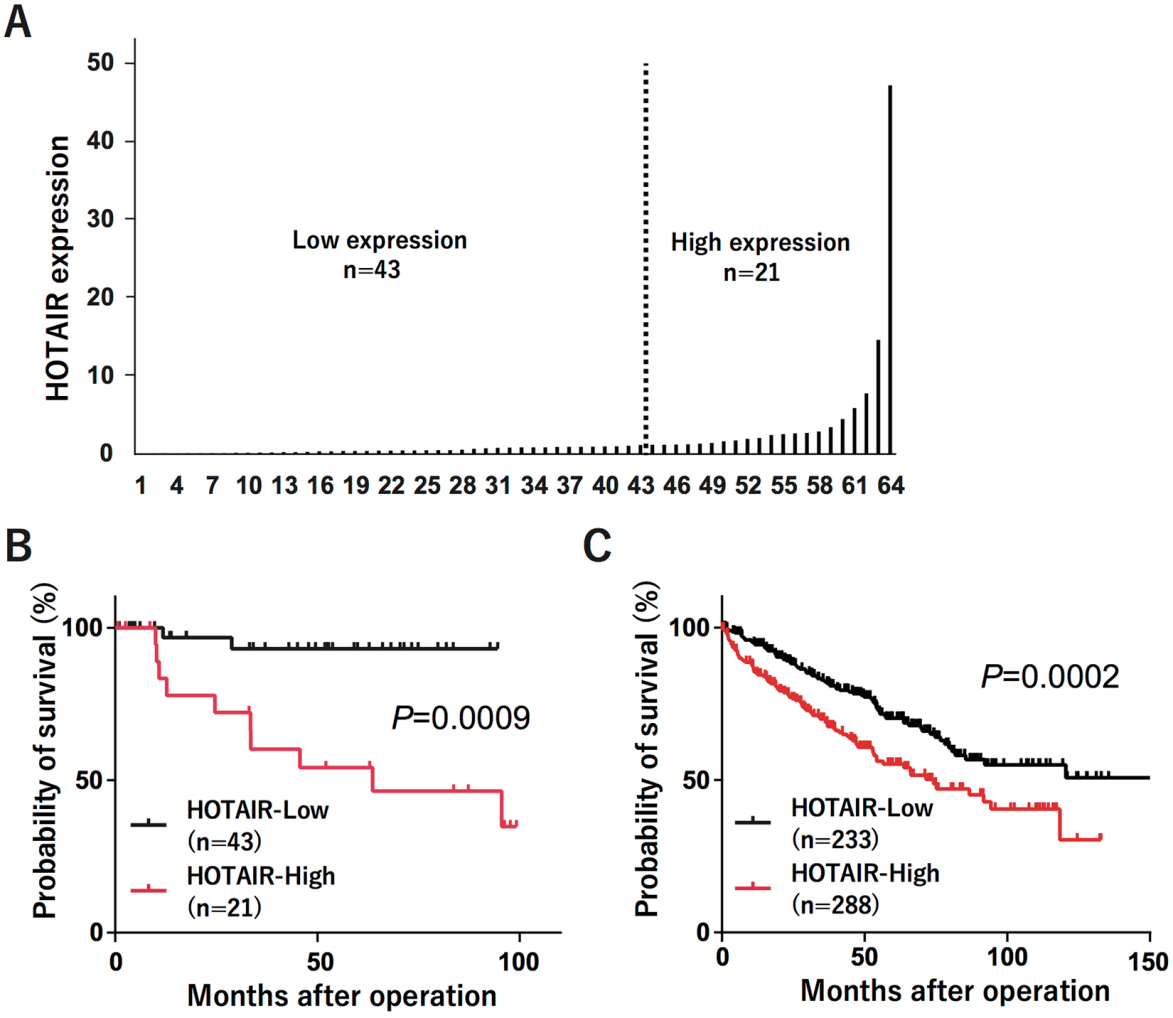
HOTAIR expression in RCCs (**A**) HOTAIR expression in 64 RCCs and their corresponding normal renal tissues was measured by quantitative real-time PCR. (**B**) Kaplan-Meier curves for cancer-specific survival according to HOTAIR expression in 64 RCC patients. (**C**) Kaplan-Meier curves for overall survival ratio according to HOTAIR expression in the RCC patient dataset obtained from TCGA (n = 521). Differences between groups were compared by log-rank test.

To evaluate correlations between HOTAIR expression and clinical characteristics, we examined the HOTAIR expression in 64 RCCs and their corresponding normal renal tissues using quantitative real-time PCR. We analyzed clinicopathological features such as age, gender, stage, T stage, N stage, M stage, nuclear grade, and vascular invasion, and measured the tumor HOTAIR expression relative to that in corresponding normal tissues. The cut-off point was determined according to the survival receiver operating characteristic (ROC) curve; tumors with HOTAIR levels at least 1.2-fold higher than that in the corresponding normal tissue were defined as high-expression, and those with HOTAIR levels below this threshold were defined as low-expression (Fig. 1A). We found that HOTAIR expression was associated with vascular invasion, nuclear grade, lymph-node metastasis, and distant metastasis (Table 1). Next, we analyzed the relationship between HOTAIR expression and patient prognosis using the Kaplan-Meier method. HOTAIR expression was significantly associated with a shorter survival in RCC patients (Fig. 1B). These results suggested that high HOTAIR expression indicates a poorer prognosis in RCC. To confirm this correlation, we searched TCGA and obtained the gene expression data and patient prognosis for 521 samples. Again, we found that high HOTAIR expression was significantly correlated with a poor prognosis (Fig. 1C). These data indicated that HOTAIR plays a critical role in RCC.

**Table 1:** HOTAIR expression and clinicopathological characteristics in RCC.

Group	HOTAIR-Low N = 43 (%)	HOTAIR-High N = 21(%)	*P*-value^a^
Age			0.787
≥60 years	29 (67)	13 (61)	
<60 years	14 (33)	8 (39)	
Sex			0.16
Male	27 (63)	17 (81)	
Female	16 (37)	4 (19)	
Types of nephrectomy			0.153
Radical	26 (60)	17 (81)	
Partial	17 (40)	4 (19)	
Fuhrman’s nuclear grade			**0.0038**
Grades 1–2	40 (93)	13 (61)	
Grades 3–4	3 (7)	8 (39)	
Vascular invasion			**0.0138**
No	31 (72)	8 (39)	
Yes	12 (28)	13 (61)	
Tumor diameter			0.057
<4 cm	19 (44)	4 (19)	
≥4 cm	24 (56)	17 (81)	
T stage			0.068
T1 + T2	35 (81)	12 (57)	
T3 + T4	8 (19)	9 (43)	
N stage			**0.0027**
N0	43 (100)	16 (76)	
N1–2	0 (0)	5 (24)	
M stage			**0.0135**
M0	39 (91)	13 (61)	
M1	4 (9)	8 (39)	
TNM stage			**0.0025**
Stage I-II	32 (74)	7 (33)	
Stage III-IV	11 (26)	14 (67)	

^a^The statistical significance of differences between two groups was estimated by Fisher’s exact test.

### Enhanced RCC-cell migration by HOTAIR *in vitro*

**Figure 2:**
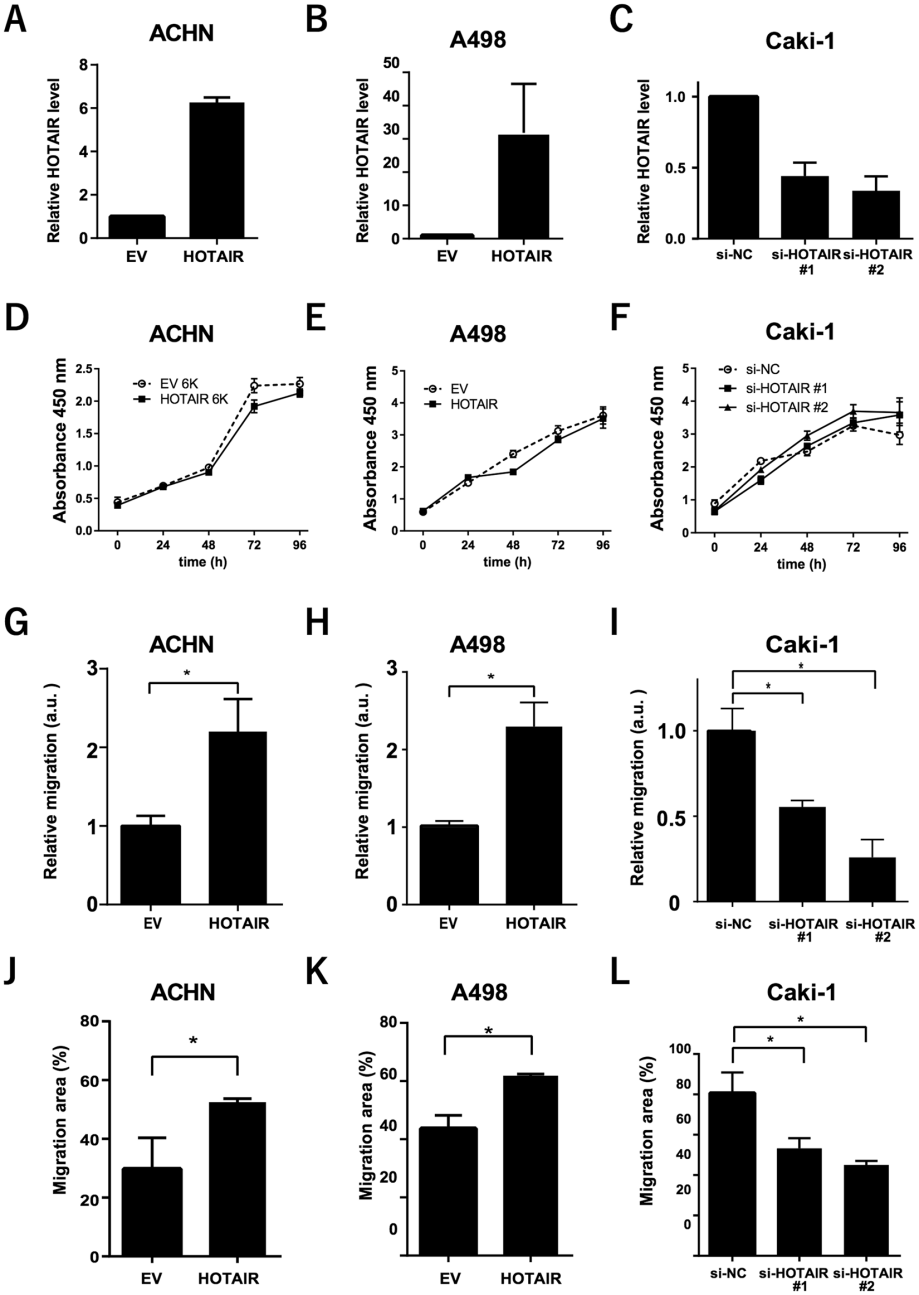
HOTAIR enhanced the migration of RCC cells *in vitro* (**A**,**B**) HOTAIR expression in ACHN and A498 cells transduced with a HOTAIR-overexpressing (HOTAIR) or empty vector (EV), determined by real-time PCR. (**C**) HOTAIR expression levels in HOTAIR-targeted siRNA-transfected Caki-1 cells, determined by real-time PCR. (**D**,**E**, and **F**) Cell-proliferation CCK-8 assays in the indicated cell lines. (**G**,**H**, and **I**) Two-chamber assays using HOTAIR-overexpressing (**G**,**H**) or knockdown cells (**I**) in arbitrary units (a.u.). (**J** to **L**) Wound-healing assays. *P < 0.05, Mann-Whitney *U* test.

Since high HOTAIR expression was significantly associated with metastasis and prognosis in patients with RCC, we used two human RCC cell lines to generate cells that stably overexpressed HOTAIR (Fig. 2A,B). These HOTAIR-transduced cells overexpressed HOTAIR at levels 6-fold higher (ACHN-HOTAIR) and 30-fold higher (A498-HOTAIR) than did empty vector (EV)–transduced cells. In Caki-1 cells, which had higher endogenous HOTAIR expression than the ACHN or A498 RCC cell lines (Supplementary Fig. S1), knocking down HOTAIR by transfection with small interfering RNA (siRNA) decreased the HOTAIR expression by 55–66% (Fig. 2C).

We assayed the effect of HOTAIR on cell proliferation using a cell-counting kit and found that the HOTAIR overexpression in ACHN and A498 cells did not alter their proliferation compared to control cells (Fig. 2D,E). HOTAIR siRNA knockdown did not affect cell proliferation in Caki-1 cells (Fig. 2F).

We evaluated HOTAIR’s role in migratory capability with a two-chamber assay and found that the migrated cells included significantly more HOTAIR-transduced than EV-transduced ACHN and A498 cells (Fig. 2G,H, and Supplementary Figure S2), and significantly fewer HOTAIR-knockdown than control Caki-1 cells (Fig. 2I). Similarly, a wound-healing assay showed that migration capacity was significantly enhanced by HOTAIR overexpression, but diminished by its knockdown (Fig. 2J–L). These data indicated that HOTAIR plays a critical role in the RCC cells’ migratory capacity.

### Effect of HOTAIR overexpression on ACHN cells *in vivo*

**Figure 3:**
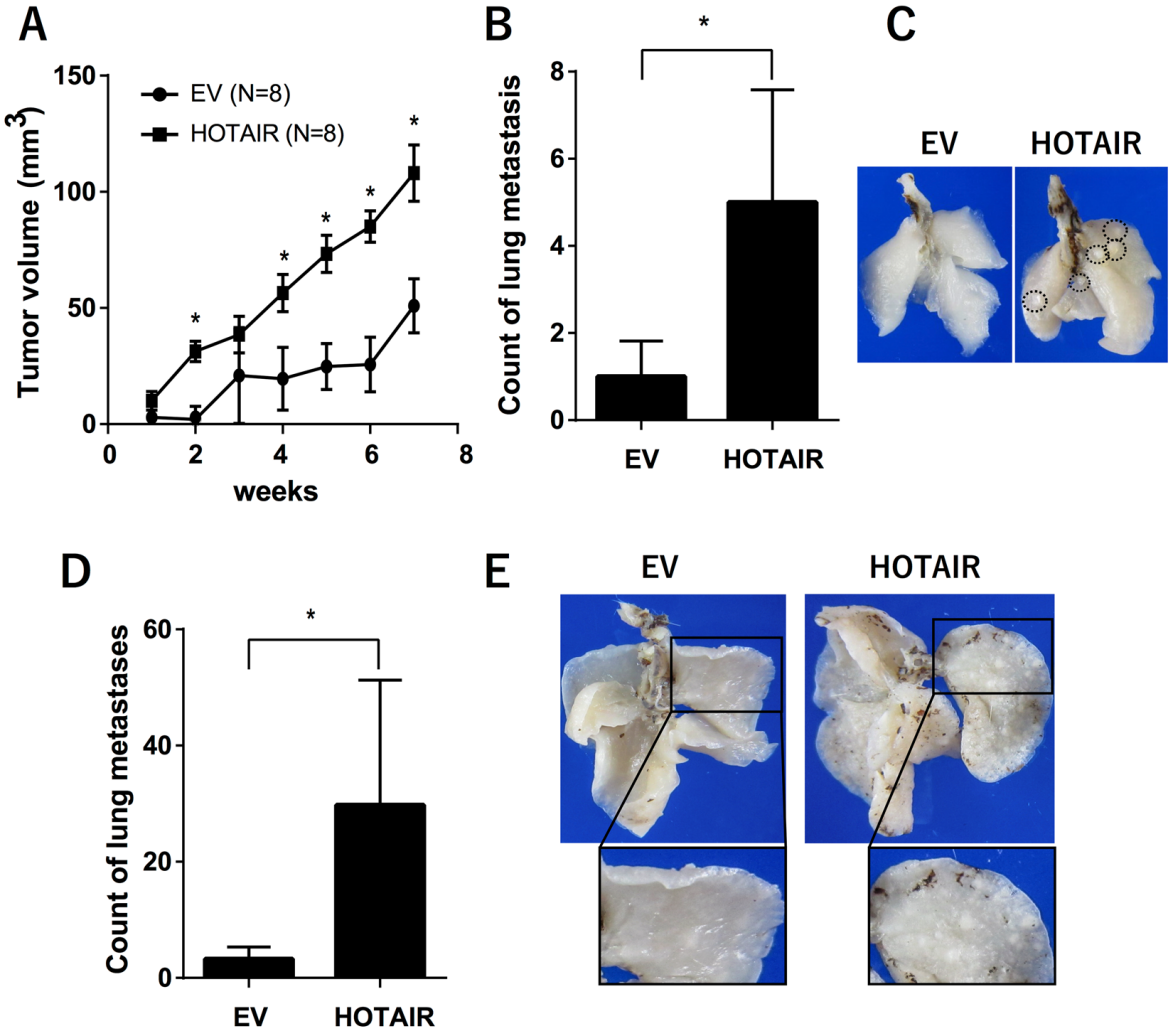
*In vivo* effects of HOTAIR overexpression in ACHN cells (**A**) Tumorigenicity was assayed by injecting HOTAIR-overexpressing or control cells subcutaneously into NOG mice; graph shows average tumor volume and standard error. (**B**) HOTAIR-overexpressing or control cells injected into the tail vein of NOG mice; 24 weeks later, metastatic pulmonary nodules were counted microscopically. (**C**) Representative images of lung metastatic nodules resulting from tail-vein injections; the dotted circles indicate metastatic foci. (**D**) Mice received orthotopic xenografts of HOTAIR-overexpressing or control cells, and pulmonary nodules were counted microscopically 14 weeks later. (**E**) Representative images of lung metastatic nodules in the orthotopic xenograft assay; the dotted circles indicate metastatic foci^*^.*P* < 0.05, Mann–Whitney *U* test.

To evaluate the malignant characteristics of HOTAIR-expressing cancer cells *in vivo*, we assayed their tumorigenicity using NOD/Shi-scid-IL2Rγ^null^ (NOG) mice. When injected subcutaneously, HOTAIR-overexpressing cells grew faster than control cells (Fig. 3A). HOTAIR overexpression in cells introduced by tail-vein injection (Fig. 3B,C) or orthotopic xenograft (Fig. 3D,E) significantly increased the number of lung metastases. These data suggested that HOTAIR overexpression enhanced the colonization and metastasis of RCC cells *in vivo*.

### Induction of IGFBP2 expression by HOTAIR

**Figure 4:**
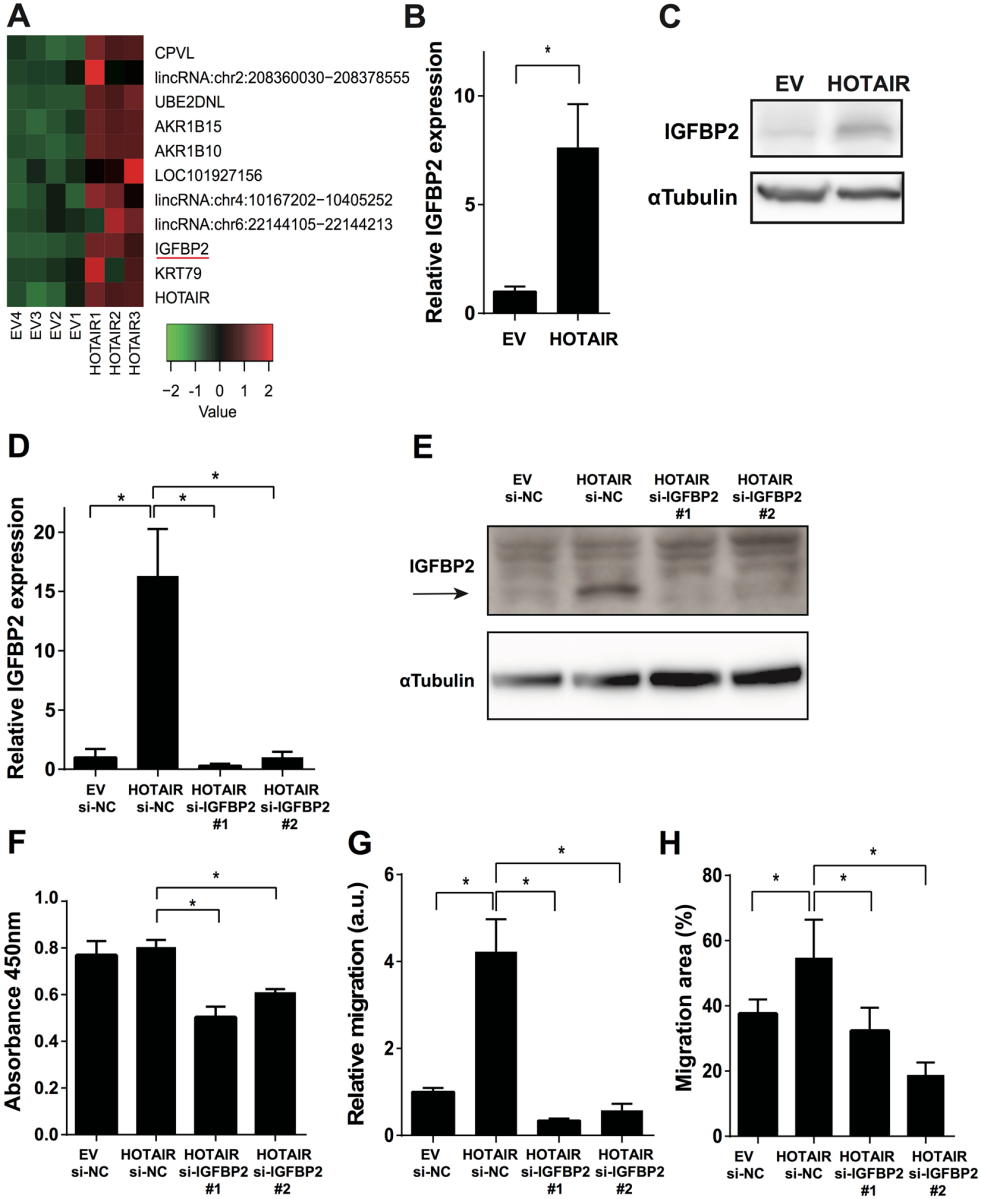
s migration in HOTAIR-expressing cells was attenuated by IGFBP2 knockdown (**A**) Heatmap showing the expression profiles of 11 genes in HOTAIR-overexpressing (HOTAIR1–3) and control (EV1–4) ACHN cells as determined by microarray analysis using the rank product algorithm. Red and green indicate higher and lower expression, respectively. (**B**, **C**) IGFBP2 expression was determined by real-time PCR (**B**) and western blotting (**C**) in HOTAIR-overexpressing ACHN cells (HOTAIR) and control cells (EV). (**D**) IGFBP2 expression was determined by real-time PCR in ACHN-EV cells transfected with control siRNA, ACHN-HOTAIR cells transfected with control siRNA, and ACHN-HOTAIR cells transfected with siRNA against HOTAIR. (**E**) IGFBP2 level in ACHN cells was measured by western blotting. (**F**) ACHN cells were transfected with the indicated siRNA, and cell proliferation was assayed 72 h later by CCK-8. (**G**, **H**) Migration measured by two-chamber assay (**G**) and wound-healing assay (**H**) in ACHN cells transfected with siRNA against IGFBP2 as indicated in (**D**); a. u., arbitrary units. *P < 0.05 by Mann-Whitney *U* test. Unprocessed original scans of blots are shown in Supplementary Figure S6.

Since our results showed that HOTAIR regulates cancer invasiveness and metastasis both *in vitro* and *in vivo*, we identified invasiveness-related genes that were upregulated in our microarray data for HOTAIR-overexpressing cells and were reported to be potentially oncogenic. After a search of the literature for each of the 11 genes that were upregulated most strongly in HOTAIR-overexpressing cells, as identified by the rank product algorithm, we chose IGFBP2 for further analysis because it has been identified as a key factor in malignancy, especially in cancer invasiveness (Fig. 4A)^17–19^. Western blots and real-time PCR results confirmed that IGFBP2 was upregulated in the ACHN-HOTAIR cells (Fig. 4B,C). Together, these results suggested that IGFBP2 acts downstream of HOTAIR in RCC.

### Attenuation of the enhanced migration of HOTAIR-overexpressing cells by IGFBP2 knockdown

To test whether IGFBP2 regulates the migratory ability downstream of HOTAIR, we transfected ACHN-HOTAIR cells with siRNAs to knockdown the *IGFBP2* gene, and confirmed that the IGFBP2 expression was significantly reduced in the transfected cells compared to control cells (Fig. 4D,E). Cell-proliferation assays revealed that 72 h after transfection, the proliferation was slightly reduced in the IGFBP2-knockdown cells compared to cells transfected with control siRNA (Fig. 4F). Cell migration was clearly reduced in IGFBP2-knockdown cells compared to cells transfected with control siRNA, in both the two-chamber (Fig. 4G) and scratch assay (Fig. 4H). We also tested HOTAIR-overexpressing A498 cells. As with ACHN cells, IGFBP2 was upregulated by HOTAIR overexpression in A498 cells, and the ability of these cells to migrate was dependent on IGFBP2 (Supplementary Figure S3, A to C). These results suggested that the migration of HOTAIR-overexpressing cells was mainly dependent on IGFBP2.

### Correlation of HOTAIR expression with IGFBP2 in RCC tissues

To determine whether HOTAIR expression was correlated with IGFBP2 expression in human RCC tissues, we extracted the total RNA from resected RCC tissues and measured the IGFBP2 and HOTAIR expression by real-time PCR. We found that the IGFBP2 expression was positively correlated with the HOTAIR expression in RCC tissues (Table 2, Fisher’s exact test).

**Table 2:** Correlation of the HOTAIR and the IGFBP2 expression in RCC.

N = 64	HOTAIR-Low	HOTAIR-High	*P*-value^*^
IGFBP2-Low	20 (31%)	12 (19%)	**0.04**
IGFBP2-High	11 (17%)	21 (33%)	

^*^Fisher’s exact test.

**Figure 5:**
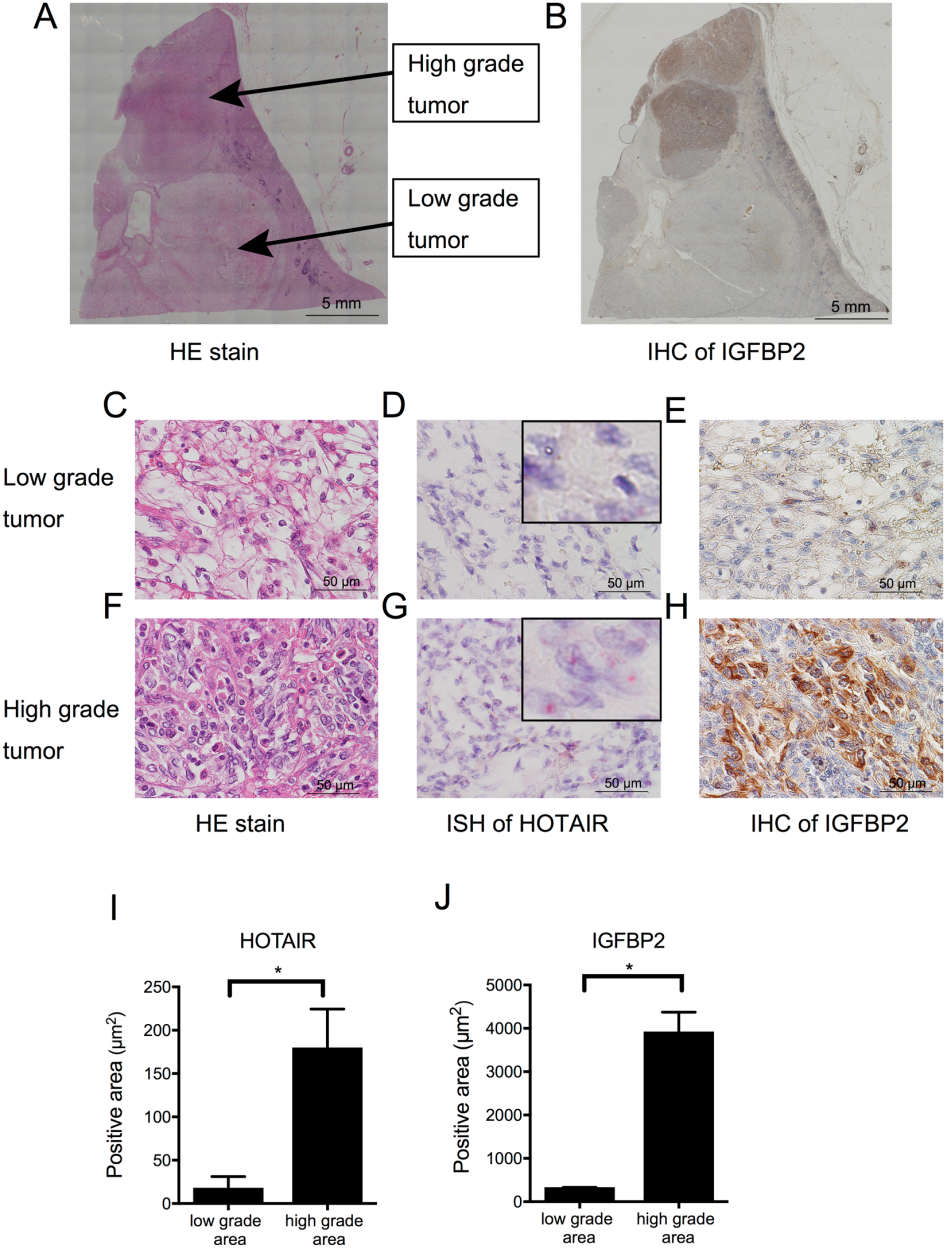
HOTAIR and IGFBP2 colocalization in high-grade tumor areas (**A**) Whole-section, low-magnification view of an HE-stained renal-cell carcinoma containing both low-grade and high-grade tumors. (**B**) Immunohistochemistry (IHC) for IGFBP2, at low magnification. (**C** to **E**) Serial sections of the low-grade tumor area at high magnification, showing HE staining (**C**), ISH for HOTAIR (**D**), and IHC for IGFBP2 (**E**). (**F** to **H**) Serial sections of the high-grade tumor area at high magnification, showing HE staining (**F**), ISH for HOTAIR (**G**), and IHC for IGFBP2 (**H**). The black arrowheads indicate dot-like positive staining of HOTAIR. (**I** and **J**) Quantification of areas positive for HOTAIR (ISH, red) and IGFBP2 (IHC, brown) using ImageJ software. Total eight areas were randomly selected from low- and high-grade tumor areas. *P < 0.001.

We used *in situ* hybridization (ISH) and immunohistochemistry (IHC) to confirm the HOTAIR and IGFBP2 co-expression in RCC tissues. Figure 5 shows representative serial sections that underwent hematoxylin and eosin (HE) staining, ISH for HOTAIR, and IHC for IGFBP2. The section contained a typical clear-cell RCC with a low nuclear grade, and another with sarcomatoid differentiation and a high nuclear grade (Fig. 5A). In the low-grade tumor area, ISH and IHC detected only slight levels of HOTAIR and IGFBP2 (Fig. 5D,E,I,J). Higher levels of HOTAIR and IGFBP2 were detected in the high-grade tumor area (Fig. 5G–J).

### IGFBP2 is a prognostic factor in RCC

**Figure 6:**
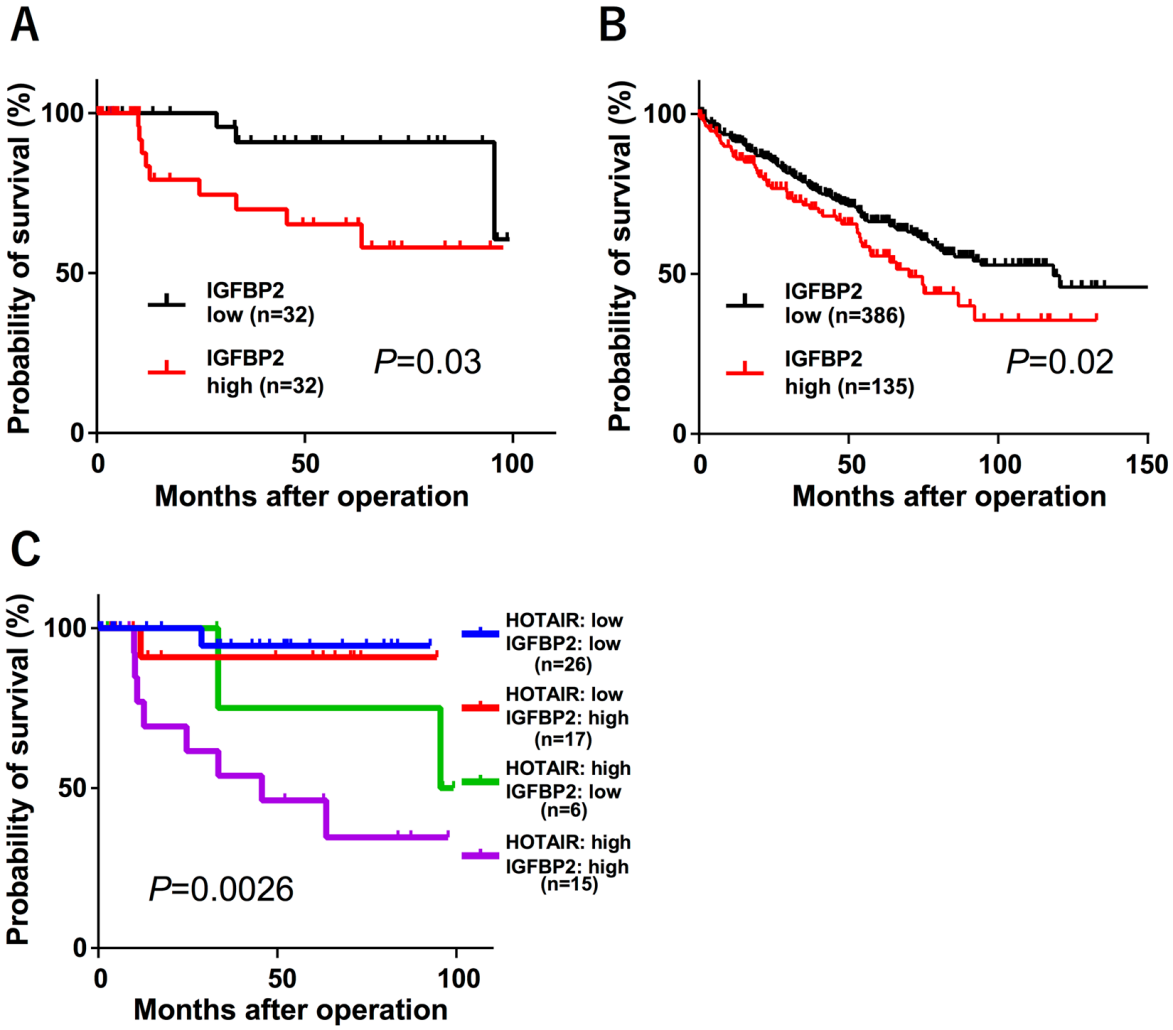
IGFBP2 is prognostic for RCC (**A**) Kaplan-Meier analysis for cancer-specific survival according to the IGFBP2 expression of in 64 RCC patients. The cut-off point was the median value in the current sample. (**B**) Kaplan-Meier curves for overall survival ratio according to IGFBP2 expression in the RCC patient dataset obtained from TCGA (n = 521). (**C**) Kaplan-Meier analysis for the cancer-specific survival rate of the four groups; differences between the groups were assessed by log-rank tests.

Finally, we investigated whether IGFBP2 was correlated with RCC prognosis. In our dataset, high IGFBP2 expression determined by real-time PCR was significantly associated with a poor prognosis (Fig. 6A). To confirm this correlation, we searched TCGA and obtained the gene expression data and patient prognosis for 521 samples. Again, we found that high IGFBP2 expression was significantly correlated with a poor prognosis (Fig. 6B). Interestingly, the group with high levels of both HOTAIR and IGFBP2 had the poorest prognosis (Supplementary Fig. S4). The prognosis for the HOTAIR^low^-IGFBP2^high^ group was almost the same as that for the HOTAIR^low^-IGFBP2^low^ group. (Fig. 6C). These data indicated that the regulation of IGFBP2 by HOTAIR is critical in RCC development.

## Discussion

In this study, we demonstrated that HOTAIR is involved in RCC. HOTAIR-overexpressing cells grew faster in an *in vivo* tumorigenicity assay but grew at a similar rate to control cells *in vitro*, suggesting that HOTAIR is involved in colonization capacity. Tail-vein injection and orthotopic xenograft assays indicated that HOTAIR induced colonization capacity in the lung. HOTAIR was associated with distant and lymph-node metastases in RCC patients. Collectively, these data suggested that HOTAIR plays critical roles in RCC’s ability to invade and colonize. A previous study showed that HOTAIR knockdown in RCC cell lines decreased their migration *in vitro* ^20^, which is compatible with our present study.

We also demonstrated that the presence of both HOTAIR and IGFBP2 in RCC is a prognostic marker in RCC, suggesting that the HOTAIR-IGFBP2 axis may represent a specific therapeutic target for RCC. Because the HOTAIR^low^-IGFBP2^high^ group had a better prognosis than the HOTAIR^high^-IGFBP2^high^ group, we speculate that HOTAIR-dependent IGFBP2 expression is important for the progression of RCC. IGFBP2 overexpression is well documented in tumors, including glioma^21^, breast cancer^22,23^, ovarian cancer^24^, prostate cancer^25^, colorectal cancer^26^, gastric cancer^27^, lung cancer^28^, leukemia^29^, and astrocytoma^30^. Recent studies implicate elevated IGFBP2 in a shorter overall survival time^31–33^. In glioma cells, IGFBP2’s interaction with integrin alpha 5 is essential for cell mobility^18^, and the IGFBP2/integrin/ILK/NF-κB signaling pathway drives cancer progression *in vivo* ^34^. Although the precise molecular mechanisms of cell invasion in RCC are unknown, IGFBP2 may be critical for this progression.

Several reports suggest that HOTAIR affects the expression of numerous genes involved in various cellular functions, especially those related to epithelial-mesenchymal transition (EMT)^7^. HOTAIR acts in esophageal squamous cell carcinoma by activating the Wnt/β-catenin signaling pathway^12^. In gastric cancer, inhibiting HOTAIR reverses the EMT process and reduces invasiveness mediated by MMP1 and MMP3^35^. In breast cancer, TGFβ1 treatment induces EMT *via* HOTAIR expression^36^. In the present study, we found that HOTAIR expression did not affect EMT-related genes (Supplementary Fig. S5). On the other hand, several reports indicate HOTAIR’s involvement in pathways unrelated to EMT. Suppressing HOTAIR upregulates the HOXA5 level and downregulates MMP2 and MMP9, which are critical players in tumor invasion and metastasis^37^. A recent report demonstrated that HOTAIR’s targets, including EMT-related genes, strongly depend on the type of cancer cell in urothelial cancer^38^. Collectively, HOTAIR primarily affects cell migration in RCC not *via* EMT signaling, but rather through an IGFBP2-related pathway, although it is not clear how HOTAIR regulates IGFBP2 expression.

Here, we newly identified IGFBP2 as a downstream molecule of HOTAIR, which is involved in the migratory capability of RCC cells. Our findings suggest that the HOTAIR-IGFBP2 axis plays critical roles in the progression of RCC, and presents a novel therapeutic target for advanced RCC treatment.

## Materials and Methods

### Ethics statement

This study was conducted according to the principles expressed in the Declaration of Helsinki and was approved by the Ethics Committees at the Miyagi Cancer Center Research Institute (Natori, Japan). Experimental protocols involving animals were approved by the Miyagi Cancer Center Animal Care and Use Committee.

### Clinical samples

Sixty-four RCC samples and their corresponding normal renal tissues were obtained from patients who underwent nephrectomy or partial nephrectomy at the Miyagi Cancer Center between 2007 and 2015. All specimens ware immediately frozen and stored at −80 °C until RNA extraction. The Institutional Review Board of the Miyagi Cancer Center approved this study protocol, and each patient provided informed, written consent. No patient received any neoadjuvant therapy, such as molecularly targeted therapy, before surgery. All tumor tissues were diagnosed pathologically as clear cell RCC. The significance of differences between two groups was estimated by Fisher’s exact test.

### Validation with TCGA data

Clinical and pathological data were manually extracted from reports downloaded from the TCGA Data Portal (https://gdc-portal.nci.nih.gov/); gene expression profiles and clinical data from 521 patients with clear cell renal carcinoma were available from the website. Tumors with HOTAIR expression of at least 0.3 FPKM (fragments per kilobase of exon per million fragments mapped) were classified as high HOTAIR, and those with less than 0.3 FPKM as low HOTAIR. Tumors with IGFBP2 expression of at least 6 FPKM were classified as high IGFBP2, and those with less than 6 FPKM as low IGFBP2. The Kaplan-Meier method for gene expression-specific survival analysis was performed using package “survival” in statistical computing language R, and the statistical significance of survival curves between different expression levels (high- and low-) was evaluated by log-rank test.

### RNA preparation, cDNA synthesis, and quantitative real-time RT-PCR

Total RNA was extracted from frozen samples and RCC cell lines by ISOGENE (Nippon Gene, Tokyo, Japan) according to the manufacturer’s protocol. For all samples, cDNAs were synthesized from 1.0 μg of total RNA with the PrimeScript 1st Strand cDNA Synthesis Kit (TaKaRa Bio, Shiga, Japan) following the manufacturer’s protocol.

HOTAIR expression was quantified as previously described^39^. The primer sequences used: for GAPDH, F 5′-TGAAGGTCGGAGTCAACGG-3′ and R 5′-AGAGTTAAAAGCAGCCCTGGTG-3′; for HOTAIR, F 5′-GGTAGAAAAAGCAACCACGAAGC-3′ and R 5′-ACATAAACCTCTGTCTGTGAGTGCC-3′; and for IGFBP2, F 5′-AGCCCAAGAAGCTGCGACCAC-3′ and R 5′-CTGCCCGTTCAGAGACATCTTGC-3′. HOTAIR expression was normalized to GAPDH expression in each sample. The HOTAIR expression in each RCC was determined relative to that in the corresponding normal renal tissue.

### Cell lines and cell culture

Three clear-cell RCC cell lines (ACHN, A498, and Caki-1) were obtained from the Japanese Cancer Research Resources Bank, Tokyo, Japan (JCRB). The cells were maintained in Dulbecco’s Modified Eagle’s Medium (DMEM, Gibco/Life technologies, Carlsbad, USA) with 10% FBS and 1% penicillin-streptomycin. The cells were incubated in a humidified incubator at 37 °C and 5% CO_2_.

### Retroviral transfection

Human HOTAIR cDNA (obtained from Addgene, Cambridge, MA, USA) was amplified by PCR and inserted into the pBabe-hygro vector (pBabe-HOTAIR). The recombinant retrovirus was produced with the Platinum-A packaging-cell line (Plat-A, kindly provided by Prof. Kitamura, Tokyo University) as described previously^40^. Briefly, Plat-A cells were transfected with pBabe-HOTAIR or a pBabe-hygro empty vector (EV). Fugene 6 (Roche Applied Science) and Opti-MEM I (Gibco/Life Technologies Co.) were added following the manufacturer’s protocol. The retrovirus-containing supernatant was collected 48 h after transfection and passed through a 0.45-μm filter. ACHN cells were infected with the recombinant retroviruses and then selected with hygromycin.

### siRNA transfection

The HOTAIR siRNAs #1 (FLJ41747HSSS160170), #2 (FLJ41747HSSS160171), the IGFBP2 siRNAs #1 (HSS142627) and #2 (HSS142629), and non-silencing control siRNA (12935–300) were purchased from Invitrogen (Carlsbad, CA, USA). The siRNA transfections were performed using Lipofectamine RNAiMAX Reagent (Life Technologies, Carlsbad, CA, USA) in antibiotic-free medium for 48 h. The efficiency of siRNA knockdown was confirmed by real-time PCR (HOTAIR and IGFBP2) and western blotting (IGFBP2).

### Cell-proliferation assay

Cell proliferation was detected with the Cell Counting Kit-8 (CCK-8) from Dojindo Laboratories (Kumamoato, Japan) according to the manufacturer’s protocol. Cells were seeded in 96-well plates (1 × 10^3^ cells per well) in normal cell-growth medium. The relative numbers of viable cells at 0, 24, 48, 72, and 96 h of incubation were assessed according to the absorbance of optical density at 450 nm detected by microplate reader (VersaMax, Molecular Devices, Sunnyvale, CA, USA).

### Two-chamber assay

Cell migration was assessed using a two-chamber assay with a cell-culture insert (8-μm pore size, BD Biosciences, San Jose, CA, USA) in 24-well plates. Cells (5 × 10^4^) were plated in each insert in serum-free medium. The bottom well contained medium with 10% FBS. After 48 h, the bottom of the insert was stained with Diff-Quick (Dade Behring Inc, Newark, DE, USA), and the cells that had invaded through the membrane to the lower surface were counted.

### Wound-healing assay

Cells were seeded in 24-well plates in normal cell-growth medium, incubated until confluent, and treated with 5 μg/ml mitomycin C (Wako, Osaka, Japan) for 3 h. A yellow pipette tip was used to make a straight scratch, simulating a wound. The medium was changed to serum-free medium. After a 24 h incubation, the area occupied by cells that had migrated into the scratch area was measured using ImageJ software.

### Western blotting

Western blotting was performed as previously described with minor modifications^39^. In brief, whole-cell lysates were prepared in RIPA buffer supplemented with a proteinase-inhibitor cocktail. The lysates were clarified by centrifugation, the supernatant was boiled, and separated by SDS-PAGE. The following antibodies were used for western blots: anti-α-tubulin (Santa Cruz Biotechnology, Santa Cruz, CA, USA, B-5–1–2) and anti-IGFBP2 (Santa Cruz Biotechnology, Santa Cruz, CA, USA, C-18).

### Animals

Six-week-old female NOG mice were purchased from the Central Institute for Experimental Animals (CIEA, Kawasaki, Japan). The mice were housed in a cleanroom at the animal care facility at Miyagi Cancer Research Center with standard temperature, humidity, and timed lighting, and given mouse chow/water ad libitum.

### *In vivo* proliferation assay by subcutaneous injection

NOG mice were given subcutaneous injections of 1.0 × 10^4^ ACHN cells transfected with a HOTAIR-overexpression (n = 8) or empty vector (n = 8) with 0.2 ml PBS. Tumors were measured weekly with a digital caliper, and tumor volume was calculated using the modified ellipsoid formula 1/2 (length × width^2^). The animals were sacrificed 8 weeks after the injection.

### *In vivo* metastasis assays by orthotopic xenograft and tail-vein injection

For *in vivo* experimental metastasis assays, 1 × 10^4^ ACHN-HOTAIR (n = 4) or ACHN-EV (n = 4) cells were injected into the tail vein of NOG mice. The mice were sacrificed 24 weeks later, and pulmonary nodules were assessed macroscopically and microscopically. The harvested organs were processed into paraffin-embedded sections, stained with HE, and examined pathologically to confirm the presence of metastatic lung tumors. In orthotopic xenograft implants, 1 × 10^5^ ACHN-HOTAIR (n = 4) or ACHN-EV (n = 3) cells in 20 μl matrigel were injected into the subcapsular area of the left kidney of the NOG mice. The mice were sacrificed 14 weeks later and the lungs were collected and analyzed. Pulmonary nodules were counted under a microscope.

### Microarray analysis

A microarray analysis was performed to search for genes regulated by HOTAIR. We used the SurePrint G3 Human GE 8 × 60 K Microarray (Agilent Technologies, Santa Clara, CA, USA) to profile the gene expression in control and HOTAIR-overexpressing ACHN cells. The obtained gene expression data were expressed in logarithmic scale, and heat maps were generated using the rank product algorithm^41^. The analyses were performed using R software. The dataset was uploaded to the Gene Expression Omnibus (GEO) database (GSE100107).

### *In situ* hybridization by RNAscope

*In situ* hybridization for HOTAIR was performed using the RNAscope 2.5 HD assay kit according to the manufacturer’s protocol (Advanced Cell Diagnostics, Hayward, CA, USA)^42^. In brief, formalin-fixed, paraffin-embedded 7-μm tissue sections were deparaffinized and pretreated with protease for hybridization with HOTAIR target mixture probes. After hybridization, a series of signal-amplification steps were hybridized to the target probes followed by color development with Fast Red. Positive staining was identified as red punctate dots in the cell. Negative control probes (DapB) and positive control probes (PPIB) were also tested for each slide to check the quality of hybridization.

### Immunohistochemistry

For IGFBP2 immunostaining, paraffin-embedded, formalin-fixed 3-μm tissue sections from human RCC patients were deparaffinized in xylene and rehydrated by washing with a series of ethanol dilutions and distilled water. Heat-induced epitope retrieval was performed by microwaving the sections in a pH-9.0 target-retrieval solution (Dako, Produktionsvej, Denmark). Endogenous peroxidases were blocked with 0.3% H_2_O_2_. The sections were incubated with anti-human IGFBP2 for 12 h (1:1,000) at 37 °C, then with mouse LINKER (Dako) for 15 min, followed by incubation with the secondary antibody and development with 3,3′-diaminobenzidine (DAB) chromogen (EnVision Detection Systems Peroxidase/DAB, Rabbit/Mouse, Dako).

### Statistical analysis

All data from both *in vitro* and *in vivo* analyses were analyzed for statistical significance by Mann-Whitney *U* test, and *P* < 0.05 was considered significant. All statistical analyses were performed using JMP 11 (SAS Institute, Cary, NC, USA) and GraphPad Prism software (Version 6.05; GraphPad Software Inc., La Jolla, CA, USA).

## Electronic supplementary material

Supplementary Information
