# Supplementary material for: Long non-coding RNA HOTAIR promotes cell migration by upregulating insulin growth factor–binding protein 2 in renal cell carcinoma
Source: Sci Rep. 2017 Sep 20;7:12016. doi: 10.1038/s41598-017-12191-z (PMC5607269; doi:10.1038/s41598-017-12191-z)
Supplement: Supplementary file 1 — Supplementary Information [file 41598_2017_12191_MOESM1_ESM.pdf]

Long non-coding RNA HOTAIR promotes cell migration by upregulating insulin growth factor–binding protein 2 in renal cell carcinoma

Hiromichi Katayama[1,6], *Keiichi Tamai[1], Rie Shibuya[1], Mao Nakamura[2], Mai Mochizuki[1], Kazunori Yamaguchi[2], Sadafumi Kawamura[3], Tatsuo Tochigi[3], Ikuro Sato[4], Takamasa Okanishi[5], Kunie Sakurai[5], Wataru Fujibuchi[5], Yoichi Arai[6], Kennichi Satoh[1]

[1]Division of Cancer Stem Cell, Miyagi Cancer Center Research Institute, Natori, Japa
[2]Division of Molecular and Cellular Oncology, Miyagi Cancer Center Research Institute, Natori, Japan
[3]Department of Urology, Miyagi Cancer Center, Natori, Japan
[4]Department of Pathology, Miyagi Cancer Center, Natori, Japan
[5]Center for iPS Cell Research and Application, Kyoto University, Kyoto, Japan
[6]Department of Urology, Tohoku University Graduate School of Medicine, Sendai, Japan

Correspondance: Keiichi Tamai
Address: 47-1, Nodayama, Medeshimashiode, Natori-city, Miyagi-ken, 981-1293, Japan
Tel: 022-384-3151
Fax: 022-381-1168
Email: tamaikeiichi@med.tohoku.ac.jp

SUPPLEMENTARY FIGURES

Supplementary Figure S1

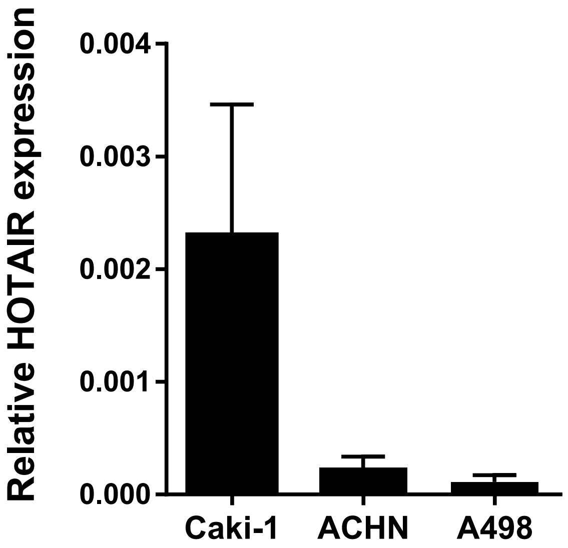

Supplementary Figure S1. HOTAIR expression in three human renal-cell carcinoma cell lines.

Supplementary Figure S2

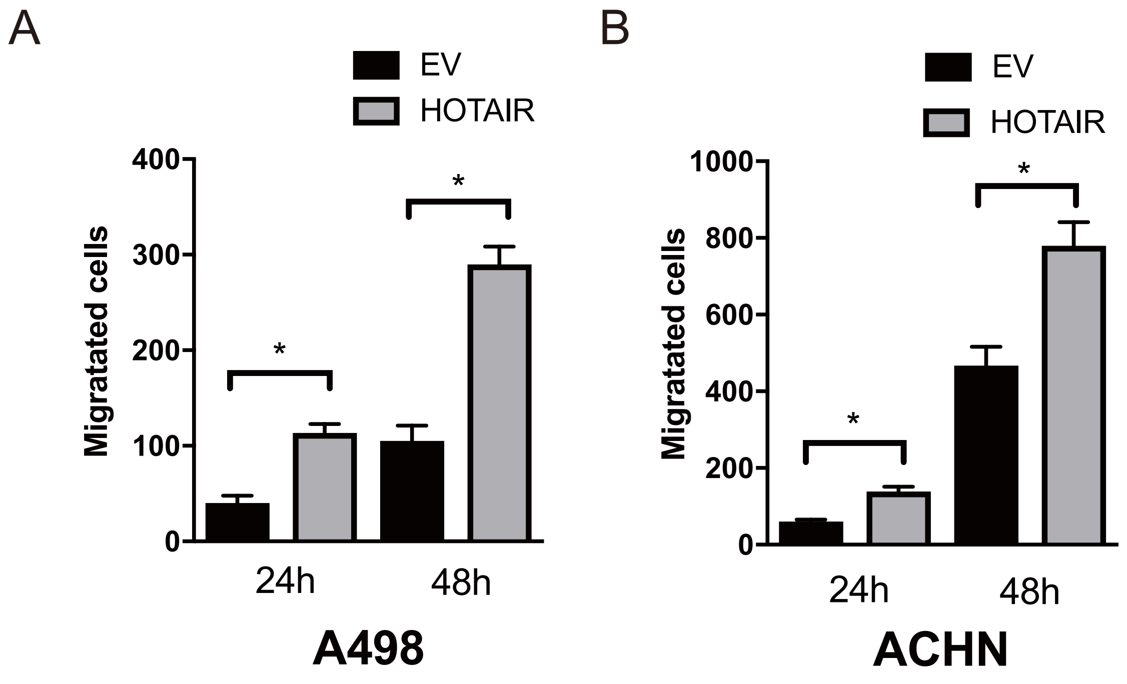

Supplementary Figure S2. Time-course analysis of trans-well migration assays using control (EV) and HOTAIR-overexpressing (A) A498 and (B) ACHN cells. *$P < 0.01$.

Supplementary Figure S3

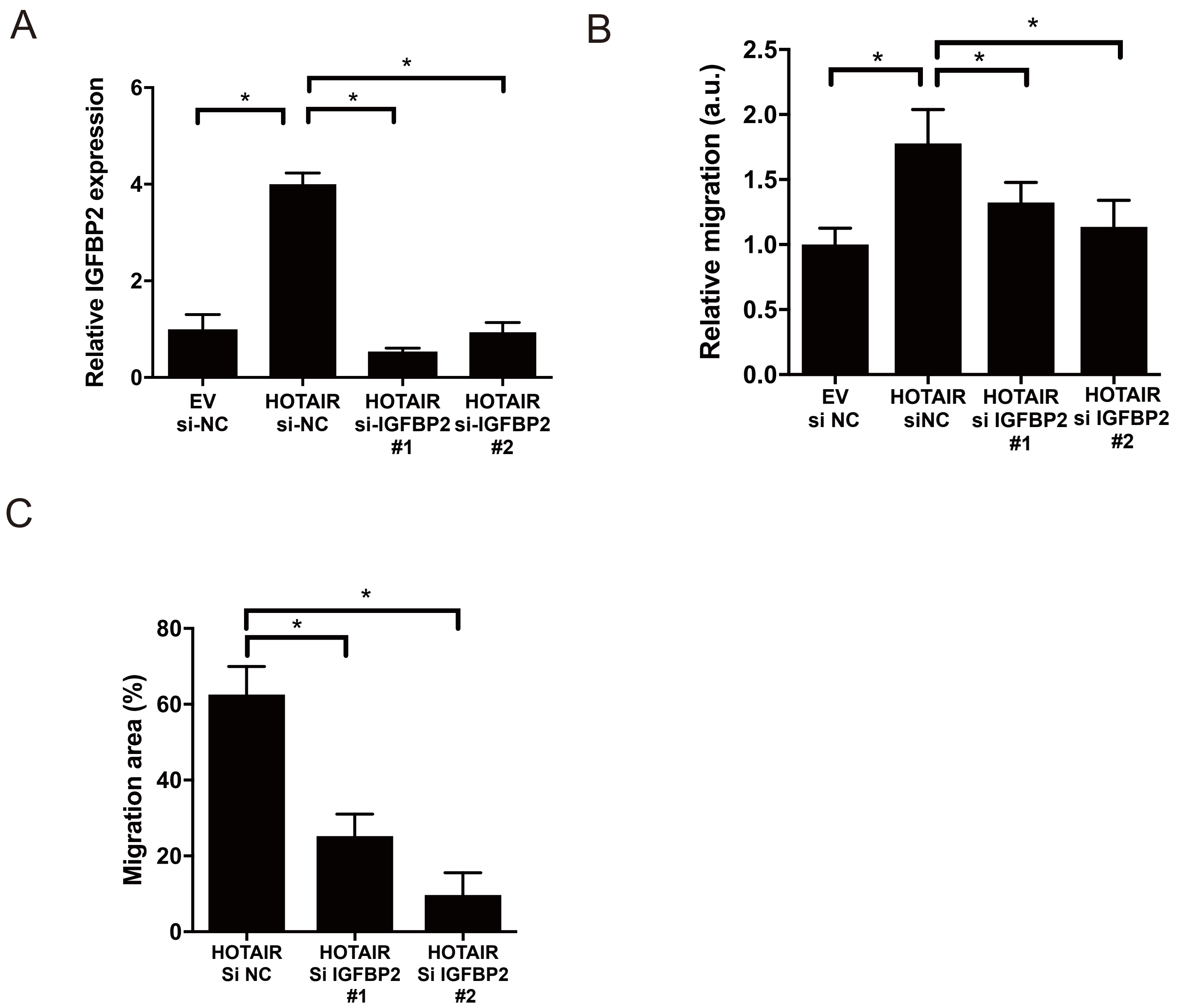

Supplementary Figure S3

Analysis of HOTAIR-overexpressing A498 cells transfected with a non-silencing control siRNA or an siRNA against HOTAIR, showing (A) IGFBP2 expression, was analyzed by real-time PCR in A498 cells., (B) Two-chamber migration assays in A498 cells. , and (C) Scratch assays. *$P < 0.01$.

Supplementary Figure S4

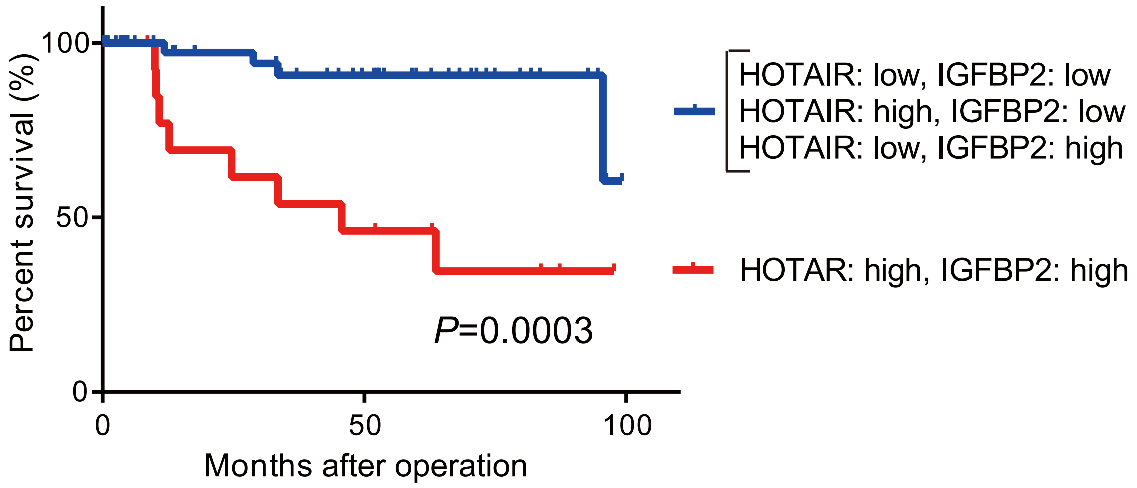

Supplementary Figure S4

Kaplan-Meier analysis for cancer-specific survival in 64 RCC patients (Fig. 6A and C) when analyzed for two groups, . The data in Fig.6 was re-analyzed for two groups (HOTAIR-high/IGFBP2-high and all other populations). Differences between these two groups were assessed by log-rank tests.

Supplementary Figure S5

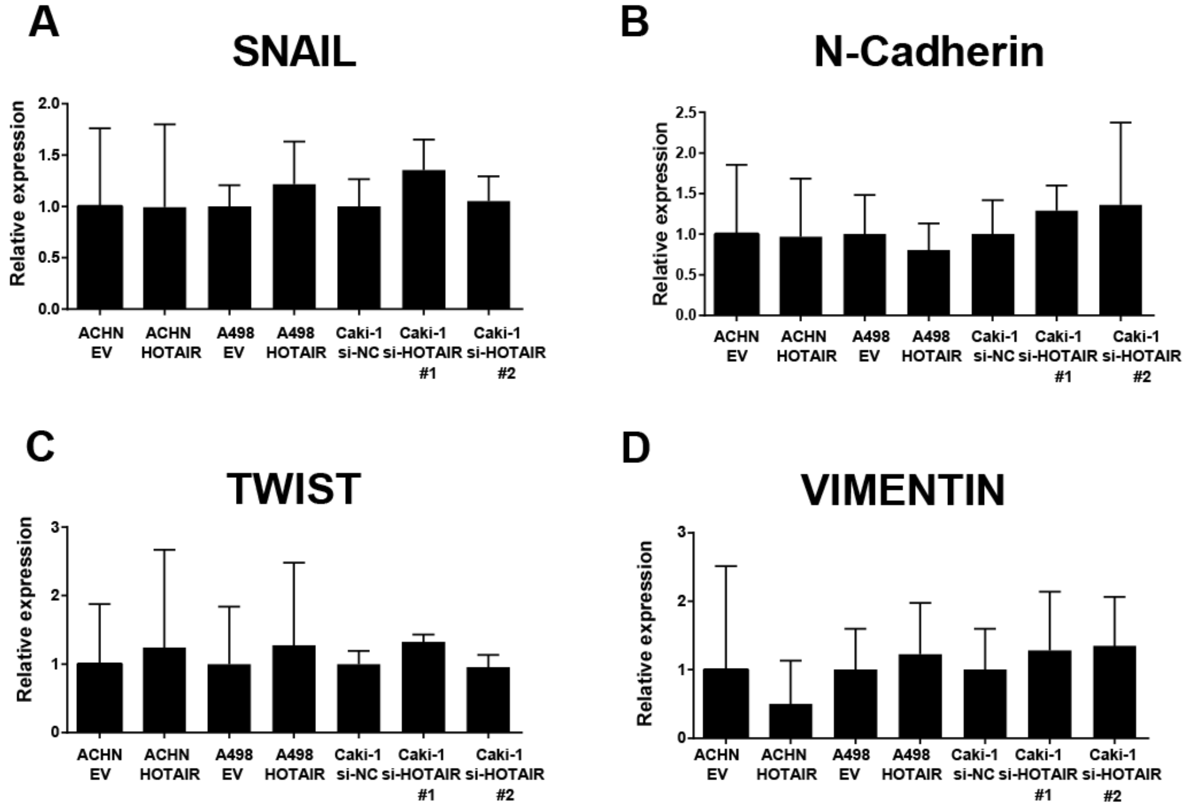

Supplementary Figure S5. Real-time PCR analysis showing the expression of EMT-associated genes in HOTAIR-overexpressing and control RCC cells. EV, empty vector.

Supplementary materials and methods

Real-time PCR

Real-time PCR was performed as described in main text. The primer sequences used: for SNAIL, F 5'- GCTGCAGGACTCTAATCCAGA-3' and R 5'- ATCTCCGGAGGTGGGATG-3'; for N-Cadherin, F 5'- CTCCATGTGCCGGATAGC-3' and R 5'- CGATTTCACCAGAAGCCTCTAC-3'; for TWIST, F 5'- AGAAGTCTGCGGGCTGTG-3' and R 5'- TCTGCAGCTCCTCGTAAGACT-3'; for VIMENTIN, F 5'- GTTTCCCCTAAACCGCTAGG-3' and R 5'- AGCGAGAGTGGCAGAGGA-3'. Expression of each gene was normalized to GAPDH expression.

Supplementary Figure S6

4C

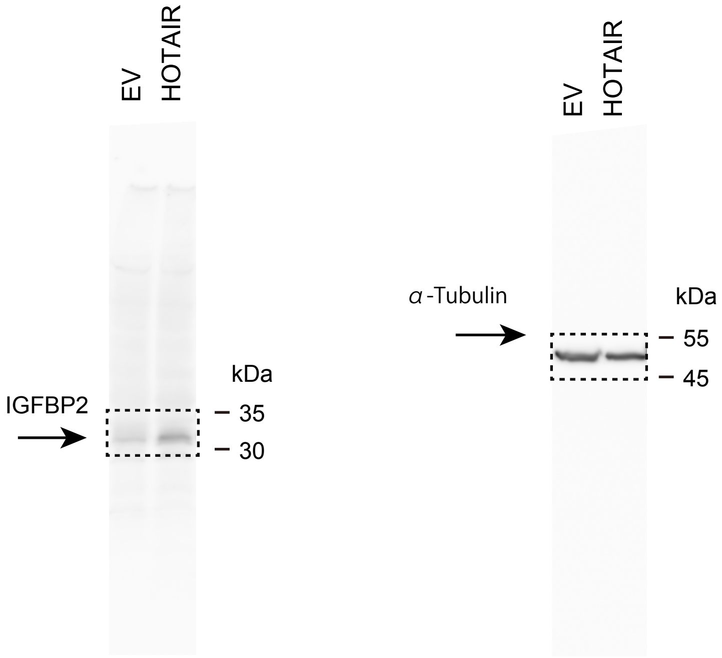

4E

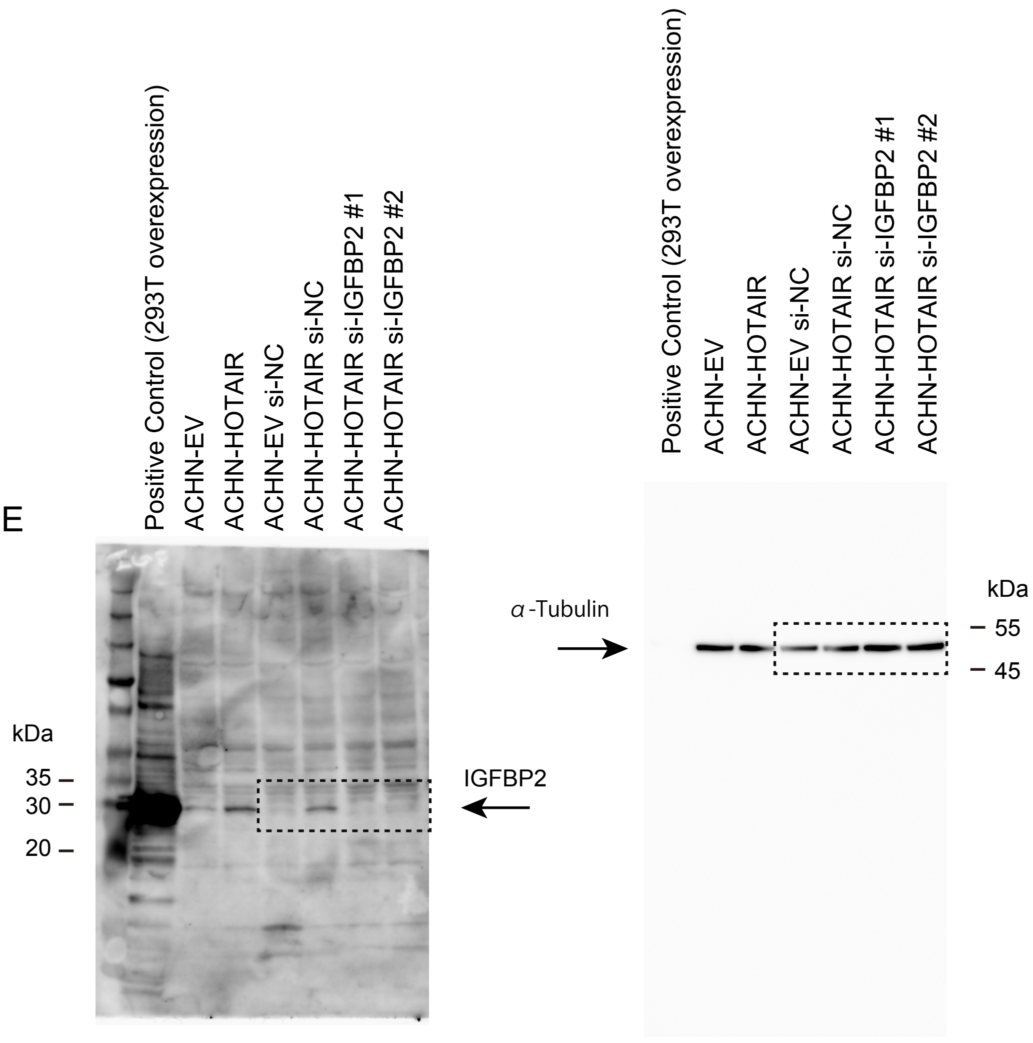

Supplementary Figure S6. Full size images of blots are shown.
